# Ultrasound Evaluation of Lower-Limb Arterial Morphology and Hemodynamics in Type 2 Diabetes Mellitus Versus Non-Diabetic Controls: A Systematic Review and Meta-Analysis

**DOI:** 10.3390/jcm15197580

**Published:** 2026-09-29

**Authors:** Salahaden R. Sultan, Faisal Albin Hajji, Muyaser Fatani

**Affiliations:** 1 Department of Radiologic Sciences, Faculty of Applied Medical Sciences, King Abdulaziz University, Jeddah 21589, Saudi Arabia; 2 Department of Radiology, King Abdulaziz University Hospital, King Abdulaziz University, Jeddah 21589, Saudi Arabia

**Keywords:** type 2 diabetes mellitus, ultrasound, lower-limb arteries, morphology, hemodynamics, peripheral arterial disease

## Abstract

**Background:** Vascular ultrasound enables non-invasive assessment of lower-limb arterial morphology and hemodynamics and may identify vascular abnormalities associated with type 2 diabetes mellitus (T2DM). This study aimed to determine whether ultrasound-derived morphological and hemodynamic parameters differ between patients with T2DM and non-diabetic controls. **Methods:** PubMed, Web of Science, EMBASE, and the Cochrane Library were systematically searched for studies published within the preceding 10 years up to June 2026. Eligible studies were original publications that quantitatively compared lower-limb arterial morphology and/or hemodynamics between adults with T2DM and non-diabetic controls using ultrasound imaging. Four studies comprising 708 participants and 2069 artery-level assessments were included. Ultrasound parameters were meta-analyzed using random-effects models, and between-group differences were evaluated using standardized mean differences (SMDs). **Results:** The pooled analyses demonstrated no statistically significant differences between patients with T2DM and non-diabetic controls in either morphological (SMD 0.23, 95% CI −0.14 to 0.61; *p* = 0.227) or hemodynamic parameters (SMD −0.14, 95% CI −0.36 to 0.09; *p* = 0.227). Heterogeneity was observed in both the morphological and hemodynamic analyses (I^2^ = 93% and 83%, respectively). **Conclusions:** Based on the available evidence, the pooled analyses identified no statistically significant overall differences in lower-limb arterial morphological or hemodynamic parameters between patients with T2DM and non-diabetic controls. The available evidence is limited by heterogeneous imaging methods and correlated artery-level measurements. Prospective multicentre studies using standardized imaging protocols and segment-specific analyses are required to clarify the clinical value of ultrasound for detecting early lower-limb vascular dysfunction in T2DM.

## 1. Introduction

Diabetes mellitus is a chronic, progressive metabolic disorder characterized by elevated blood glucose levels resulting from impaired insulin secretion and action [1]. According to the Global Burden of Diseases (GBD) 2019, diabetes mellitus ranked as the eighth leading cause of combined death and disability worldwide, affecting nearly 460 million individuals across all age groups and regions in 2019 [2]. The International Diabetes Federation estimates that 537 million people were living with diabetes globally in 2021, generating healthcare expenditures of approximately US$966 billion—an amount projected to exceed $1054 billion by 2045 [3]. These suggest that diabetes imposes a significant and growing burden on healthcare systems [4].

Type 2 diabetes mellitus (T2DM) is a well-established independent risk factor for cardiovascular disease, increasing the risk of coronary arterial disease, stroke, and peripheral arterial disease (PAD) [5]. Insulin resistance, a central feature of T2DM, promotes increased circulating free fatty acids, formation of advanced glycation end-products, activation of protein kinase C, oxidative stress, and endothelial dysfunction [6]. These initiate a vascular inflammatory response characterized by endothelial leukocyte infiltration, macrophage proliferation, impaired vasodilatation, and progressive arterial injury [7]. These accelerate atherosclerosis and increase the risk of macrovascular events [6]. T2DM is also associated with lower-limb ischaemic complications and an increased likelihood of revascularisation or amputation [8]. Although cardiovascular complications in T2DM may decline with improved risk-factor management and greater use of therapies that control blood glucose, promote weight loss, and reduce cardiovascular risk [6,9], early detection of subclinical lower-limb vascular abnormalities remains essential for preventing cardiovascular and limb-related complications.

The ankle-brachial pressure index (ABPI) is a non-imaging tool for detecting significant PAD [10]. An ABI ≤ 0.9 significantly increases the risk of all-cause mortality, cardiovascular mortality, and major cardiovascular events across all Framingham Risk Score categories [11]. The 2016 AHA/ACC guidelines recommend resting ABI testing for patients with symptoms or examination findings suggestive of PAD and consider it reasonable for those at increased risk without clear symptoms [12]. In cases of non-compressible arteries (ABPI > 1.3), the toe-brachial index is preferred, and for symptomatic patients with normal resting studies, exercise ABI testing may reveal abnormalities in up to 30% of cases [13,14]. The ABPI has high specificity but limited sensitivity for detecting peripheral arterial disease (PAD) in individuals with diabetes mellitus [15]. Arterial calcification and vessel non-compressibility may falsely elevate ankle pressures, resulting in normal ABPI values despite clinically significant PAD [16]. Accordingly, the 2024 ACC/AHA guideline emphasizes that ABPI sensitivity is reduced in diabetes and recommends additional physiological assessment when ABPI measurements are unreliable or clinical suspicion persists [17]. A normal ABPI should therefore not preclude further investigation in patients with diminished pedal pulses, neuropathy that may mask ischaemic symptoms, diabetic foot ulceration, tissue loss, arterial calcification or longstanding T2DM with complications. These highlight the importance of integrating ABPI with complementary vascular assessments and imaging modalities to improve PAD detection in this population.

Vascular ultrasound is a well-established, non-invasive imaging modality that can assess the anatomical distribution of arterial disease. Ultrasound imaging utilizes non-ionizing high-frequency sound waves to generate real-time images of internal structures, enabling morphological evaluation. In addition to visualizing structural abnormalities, ultrasound Doppler imaging obtained from peripheral arteries provides valuable information on hemodynamics, offering a comprehensive vascular assessment [18]. In a cohort of 324 adults with T2DM, duplex ultrasonography detected PAD in 28.7% of participants, compared with 5.6% using ABPI. Notably, 77 participants had duplex-confirmed PAD despite ABPI values of 0.91–1.40, with most lesions occurring in the infrapopliteal arteries, consistent with a distal and multisegmental pattern of PAD associated with diabetes [19].

Despite the established vascular effects of T2DM and the increasing clinical use of non-invasive ultrasound for vascular evaluation, it remains unclear whether ultrasound morphological and hemodynamic parameters differ between patients with T2DM and non-diabetic controls. Therefore, the aim of the present study was to systematically review and meta-analyze studies on the use of ultrasound imaging to evaluate lower-limb arteries in patients with T2DM and non-diabetic controls.

## 2. Methods

### 2.1. Search Strategy and Study Selection

This study was conducted according to the Preferred Reporting Items for Systematic Reviews and Meta-Analyses (PRISMA) guidelines (Table S1) [20]. The review protocol was not prospectively registered. Web of Science, EMBASE, and the Cochrane Library databases were systematically searched for studies published within the last 10 years up to June 2026 that evaluated lower-limb arteries in diabetic patients and non-diabetic controls using ultrasound Doppler imaging. Search terms used in titles and abstracts included the following search-words: diabetes mellitus OR diabetic foot OR diabetic OR type 2 diabetes OR healthy volunteers OR non-diabetic controls OR healthy controls OR control group OR control groups OR healthy participants AND doppler ultrasound OR duplex ultrasound OR doppler ultrasonography OR colour doppler OR color doppler OR spectral doppler OR vascular ultrasound OR arterial ultrasound AND peak systolic velocity OR end-diastolic velocity OR resistive index OR pulsatility index OR acceleration time OR intima-media thickness OR flow volume OR arterial diameter OR blood flow velocity OR blood flow volume OR ankle brachial index OR ankle-brachial index AND lower extremity OR leg OR femoral artery OR popliteal artery OR tibial artery OR lower limb OR dorsalis pedis OR posterior tibial OR anterior tibial (Table S2). The search was limited to studies involving humans, adults aged ≥ 19 years, and articles published in English. English-language titles and abstracts were used for initial screening. Studies with non-English full texts remained eligible for assessment and data extraction, and no study identified by the search was excluded because of its publication language. Predefined inclusion and exclusion criteria were applied to minimize selection bias. Eligible studies included original published articles that used ultrasound imaging parameters to compare morphological changes and hemodynamics of lower-limb arteries in T2DM patients and non-diabetic controls. Non-original articles, including reviews, study protocols, conference abstracts, pictorial essays, editorials, and viewpoints, were excluded. Studies that did not evaluate lower-limb arteries using ultrasound imaging, did not provide quantitative ultrasound parameters on lower-limb arteries in diabetic patients and non-diabetic controls, or assessed lower-limb arteries in T1DM or post-intervention procedures were also excluded. All three authors participated in study screening and data extraction. Any disagreements regarding study eligibility or extracted information were resolved through discussion and consensus.

### 2.2. Data Acquisition

From the included studies, the following data were extracted: study reference, primary study aim, patient demographic and clinical characteristics, sample size, ultrasound Doppler used, artery assessed, and study findings. For the meta-analysis, quantitative B-mode and Doppler ultrasound parameters were compared between diabetic patients with lower-limb arterial disease against non-diabetic control groups. Quantitative B-mode and Doppler ultrasound imaging parameters included measurements of vessel wall thickness, diameter, inner radius, peak systolic velocity (PSV), end-diastolic velocity (EDV), mean velocity (MV), pulsatility index (PI), and resistance index (RI), and flow volume. The number of arteries evaluated was considered the sample size. Mean and standard deviation values of B-mode and Doppler parameters for diabetic and non-diabetic groups were extracted for analysis. If a study reported multiple ultrasound parameters from the same group, the group sample size was divided by the number of parameters to avoid double-counting participants in the same forest plot.

### 2.3. Quality and Risk of Bias

Quality assessment of the included studies was assessed using 11 criteria from the Quality Assessment of Diagnostic Accuracy Studies (QUADAS) [21]. The assessment considered the clarity of participant-selection criteria; availability of clinical and demographic information of recruited participants; reporting the duration of diabetes mellitus since diagnosis; use of ABPI use of the ABPI for all participants; reporting of the time interval between ultrasound and ABPI evaluation; detailed description of the ABPI standard methodology; clear description of the ultrasound imaging protocol; blinding of image assessment or outcome evaluation; completeness of outcome data; and explanation of participant withdrawals or exclusions where applicable. Risk of bias was assessed using the ROBINS-E tool across seven domains: bias due to confounding; bias arising from measurement of the exposure; bias in the selection of participants into the study; bias due to post-exposure interventions; bias due to missing data; bias arising from measurement of the outcome; and bias in the selection of the reported result. Responses to the signaling questions were recorded as “Yes/Probably yes,” “No/Probably no” or “No information” and interpreted according to the direction of each question. Responses supporting the absence of bias informed a low-risk judgment, whereas responses indicating a potential source of bias informed a higher-risk judgment. “No information” was assigned when the available evidence was insufficient and was considered to represent a high risk of bias in the overall study-level assessment. Domain-level and overall judgments were classified as low risk, moderate risk, or high risk [22].

### 2.4. Statistical Analysis

Data were grouped before analysis into morphological and hemodynamic outcomes. Morphological outcomes included arterial wall thickness, inner radius, and diameter. Hemodynamic outcomes included flow velocity (PSV, EDV, and MV), flow volume, RI, and PI. Each outcome domain was presented in a separate forest plot and subgrouped according to the ultrasound parameter. Outcomes were pooled as standardized mean differences (SMD) with 95% confidence intervals using a random-effects model. Data from each group were analyzed as forest plots using the Cochrane Review Manager software version 5.4 (The Cochrane Collaboration, Copenhagen, Denmark; 2020). Positive SMD indicated higher values in patients with T2DM, whereas negative values indicated higher values in non-diabetic controls. Statistical heterogeneity was evaluated using I^2^ statistics. Statistical significance was defined as *p* < 0.05.

## 3. Results

### 3.1. Study Selection

**Figure 1:**
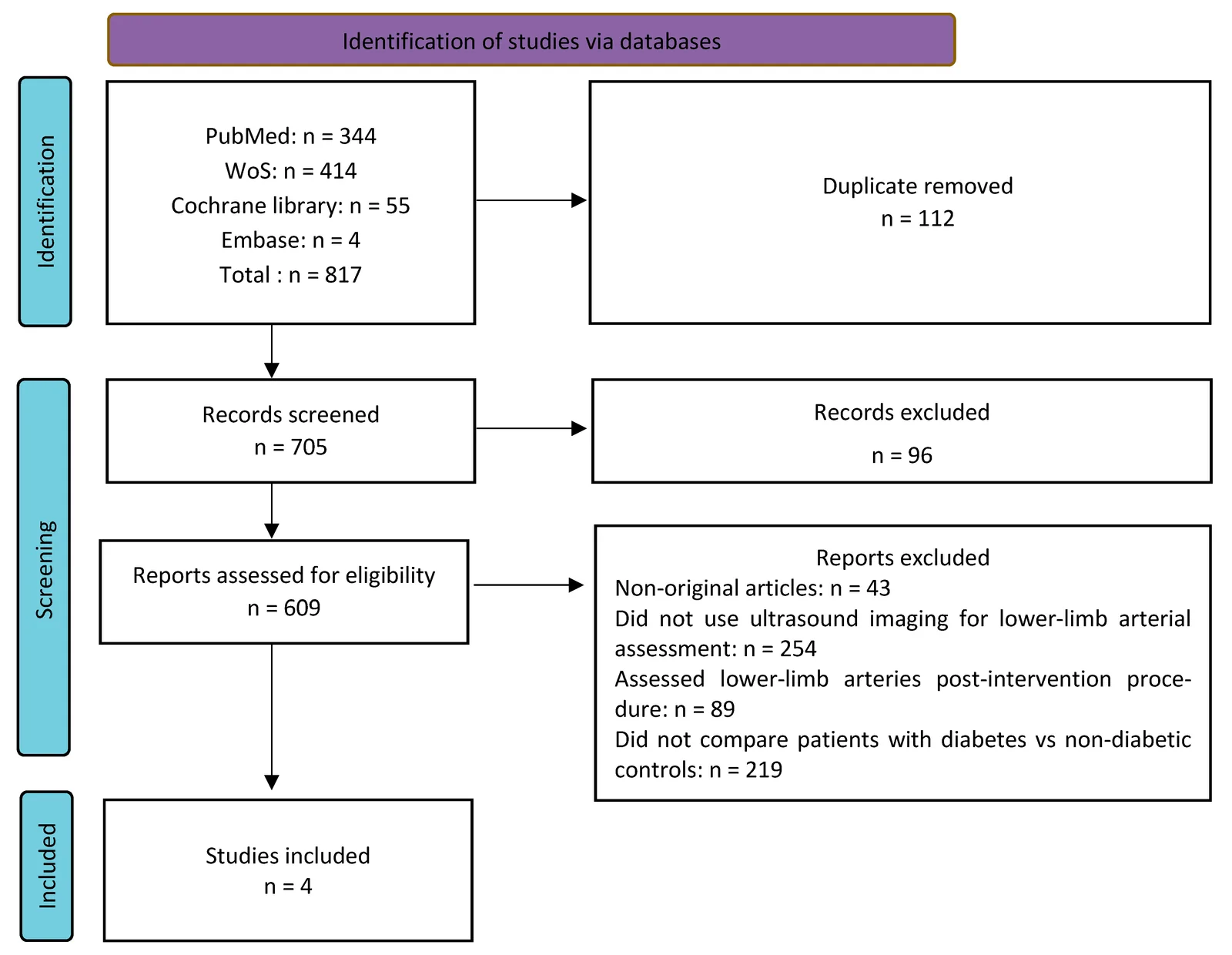
Flow chart for study retrieval and selection.

The database search identified 817 records from PubMed (n = 344), Web of Science (n = 414), the Cochrane Library (n = 55), and EMBASE (n = 4). After removing 112 duplicates, 705 records were screened, of which 96 were excluded. The full texts of 609 reports were assessed for eligibility, and 605 were excluded for the following reasons: non-original articles (n = 43), studies that did not use ultrasound imaging to assess lower-limb arteries (n = 254), studies assessing lower-limb arteries following an interventional procedure (n = 89), and studies that did not compare patients with diabetes with non-diabetic controls (n = 219). Four studies were included in the systematic review and meta-analysis (Figure 1). The included studies were published between 2017 and 2026 and comprised 708 participants: 439 patients with T2DM and 269 non-diabetic controls. Overall, 2069 artery-level assessments were reported, including 1200 in patients with T2DM and 869 in controls. The assessed vessels included the femoral artery (FA), popliteal artery (Pop A), posterior tibial artery (PTA), dorsalis pedis artery (DPA), and plantar digital artery (PDA). The characteristics and principal findings of the included studies are summarized in Table 1.

### 3.2. Morphological Assessment of Lower-Limb Arteries

**Figure 2:**
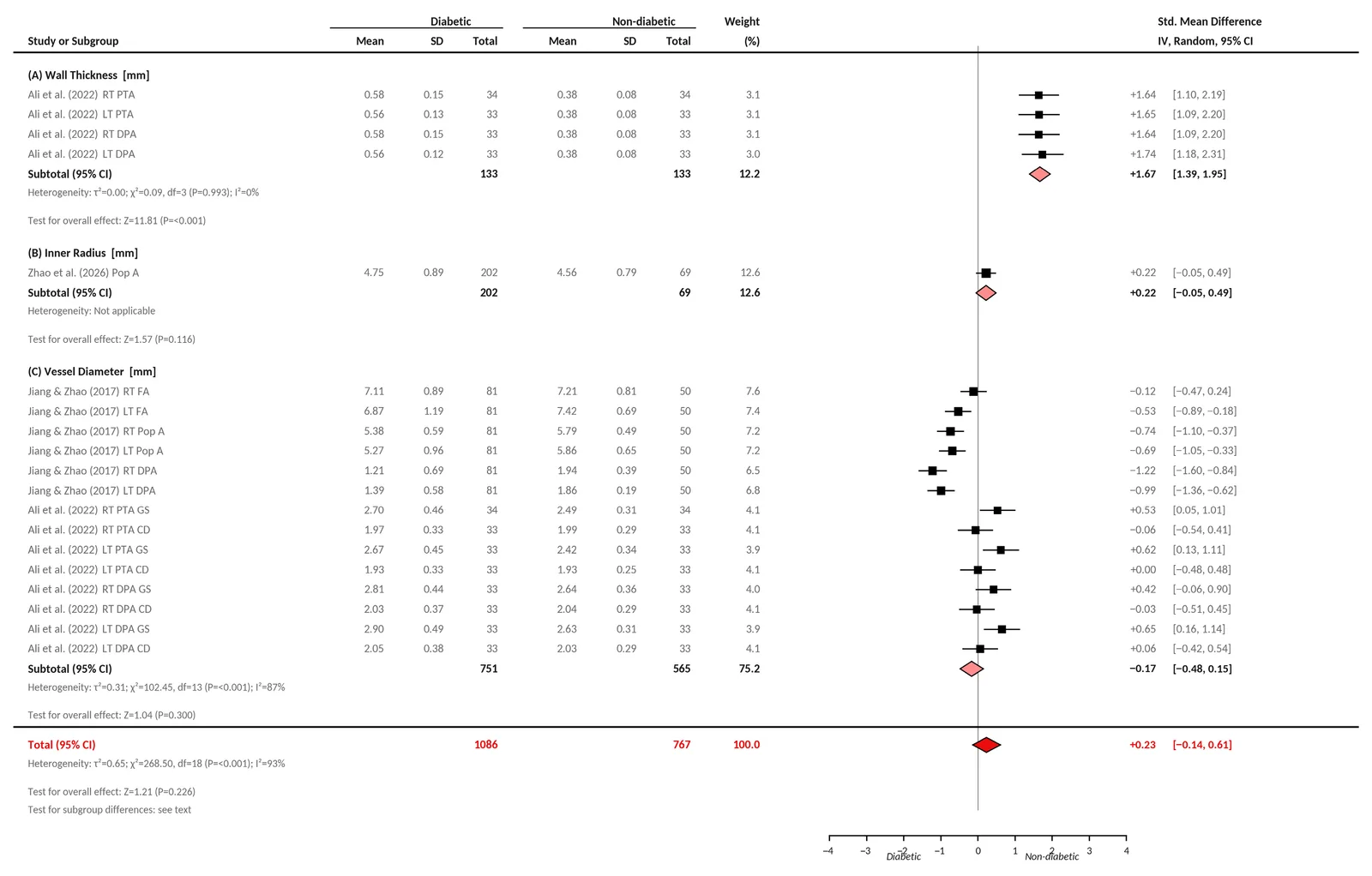
Ultrasound evaluation of lower-limb arterial morphology in patients with T2DM and non-diabetic controls [24,25,26]. CD, color Doppler; DPA, dorsalis pedis artery; FA, femoral artery; GS, grayscale; LT, left; Pop A, popliteal artery; PTA, posterior tibial artery; RT, right.

The overall pooled morphological effect was not statistically significant (SMD: 0.23, 95% CI: −0.14 to 0.61; *p* = 0.227, Figure 2), and heterogeneity was observed (I^2^ = 93%). Subgroup analysis showed that wall thickness was significantly greater in patients with T2DM than in non-diabetic controls (SMD, 1.67; 95% CI, 1.39–1.95; *p* < 0.001). In contrast, arterial inner radius did not differ significantly between the groups (SMD, 0.22; 95% CI, −0.06 to 0.49; *p* = 0.117). These findings were derived from a single study. Ali et al. (2022) [25] reported greater wall thickness in the PTA and DPA in patients with T2DM than in controls (0.56–0.58 mm vs. 0.38 mm, *p* < 0.05). Grayscale vessel diameters were also greater in the T2DM group (2.67–2.90 mm vs. 2.42–2.64 mm, *p* < 0.05), but lumen diameters measured using color Doppler did not differ significantly between groups (1.93–2.05 mm vs. 1.93–2.04 mm, *p* > 0.05) [25]. In contrast, Jiang and Zhao (2017) reported smaller arterial diameters in patients with T2DM than in control, However, statistically significant reductions were reported for the left popliteal artery (5.27 vs. 5.86 mm) and PDA (1.21–1.39 vs. 1.86–1.94 mm; all *p* < 0.05), whereas FA diameter (6.87–7.11 mm in T2DM versus 7.21–7.42 mm in controls) and right Pop A diameter (5.38 mm in T2DM versus 5.79 mm in controls) showed no statistical difference between groups (*p* > 0.05) [24]. Zhao et al. (2026) reported a non-significant difference in Pop A inner radius in T2DM patients than in controls (*p* > 0.05) [26]. Ultrasound morphological parameters are presented in Table 2.

### 3.3. Hemodynamic Assessment of Lower-Limb Arteries

**Figure 3:**
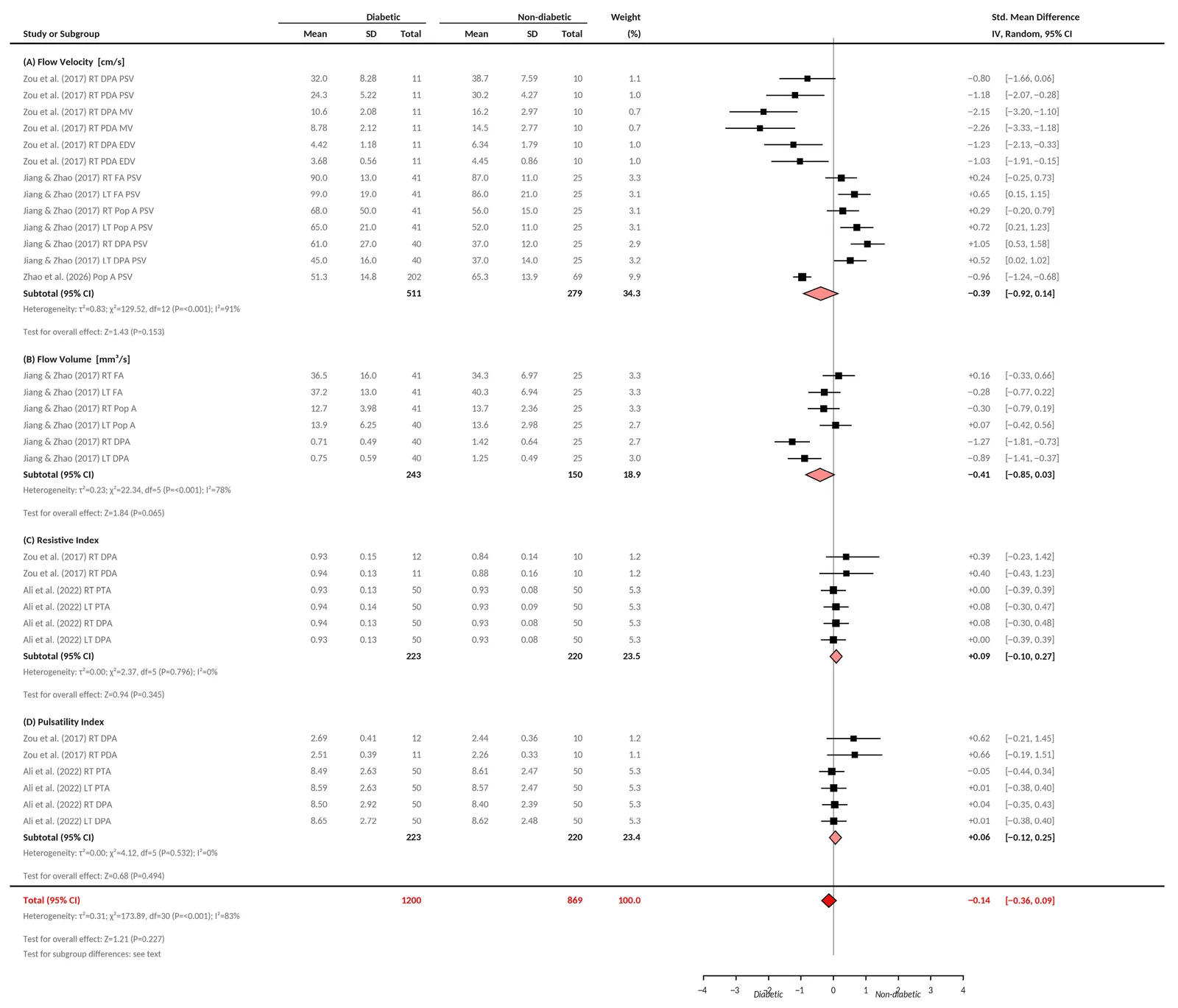
Ultrasound evaluation of lower-limb arterial hemodynamics in patients with T2DM and non-diabetic controls [23,24,25,26]. DPA, dorsalis pedis artery; EDV, end-diastolic velocity; FA, femoral artery; LT, left; MV, mean velocity; PDA, plantar digital artery; PI, pulsatility index; Pop A, popliteal artery; PSV, peak systolic velocity; PTA, posterior tibial artery; RI, resistive index; RT, right.

The overall pooled hemodynamic effect did not differ significantly between patients with T2DM and non-diabetic controls (SMD: −0.14, 95% CI: −0.36 to 0.09; *p* = 0.227, Figure 3), with substantial overall heterogeneity (I^2^ = 83%). The pooled flow-velocity estimate was lower in patients with T2DM than in non-diabetic controls; however, the difference was not statistically significant (SMD: −0.39, 95% CI: −0.92 to 0.14; *p* = 0.151). Flow volume was also lower in patients with T2DM, although the difference did not reach statistical significance (SMD: −0.41, 95% CI: −0.85 to 0.03; *p* = 0.065; I^2^ = 77%). No significant differences were observed between the groups in RI (SMD: 0.09, 95% CI: −0.10 to 0.27; *p* = 0.364; I^2^ = 0%) or PI (SMD: 0.06, 95% CI: −0.13 to 0.25; *p* = 0.522; I^2^ = 0%). Zou et al. (2017) [23] reported significantly lower PSV in patients with T2DM than in controls in both the DPA (32.02 ± 8.28 vs. 38.67 ± 7.59 cm/s, *p* < 0.05) and PDA (24.33 ± 5.22 vs. 30.20 ± 4.27 cm/s, *p* < 0.05). MV was also lower in the DPA (10.55 ± 2.08 vs. 16.24 ± 2.97 cm/s, *p* < 0.05) and PDA (8.78 ± 2.12 vs. 14.54 ± 2.77 cm/s, *p* < 0.05), as was EDV (4.42 ± 1.18 vs. 6.34 ± 1.79 cm/s and 3.68 ± 0.56 vs. 4.45 ± 0.86 cm/s, *p* < 0.05, respectively) [23]. Similarly, Zhao et al. (2026) observed lower Pop A PSV in T2DM (51.31 ± 14.81 vs. 65.35 ± 13.94 cm/s), reporting a threshold of ≤61.90 cm/s demonstrated 79% sensitivity and 30% specificity [26]. Conversely, Jiang and Zhao (2017) reported higher PSV in DPA (0.45–0.61 m/s in T2DM vs. 0.37 m/s in control, *p* < 0.05) and lower flow volume in T2DM than in control (0.71–0.75 mm^3^/s vs. 1.42–1.25 mm^3^/s, *p* < 0.05) [24]. In contrast, flow volumes in the proximal arteries overlapped between groups: FA values ranged from 36.49 to 37.21 mm^3^/s in T2DM and from 34.29 to 40.31 mm^3^/s in controls (*p* > 0.05), while Pop A values ranged from 12.65 to 13.95 and from 13.58 to 13.70 mm^3^/s (*p* > 0.05), respectively [24]. Regarding waveform indices, Zou et al. (2017) observed higher RI and PI values in the DPA and PDA in T2DM than in controls (RI: 0.93 ± 0.15 vs. 0.84 ± 0.14 and 0.94 ± 0.13 vs. 0.88 ± 0.16, *p* < 0.05; PI: 2.69 ± 0.41 vs. 2.44 ± 0.36 and 2.51 ± 0.39 vs. 2.26 ± 0.33, *p* < 0.05, respectively) [23]. In contrast, Ali et al. (2022) found no significant difference in RI values between groups (0.93–0.94 in T2DM vs. 0.93 in controls, *p* > 0.05), nor PI (8.49–8.65 vs. 8.40–8.62, *p* > 0.05) [25]. Ultrasound hemodynamic parameters are presented in Table 3.

### 3.4. Quality and Risk of Bias

All four studies provided clinical and demographic information and described the ultrasound imaging protocol and equipment used and participant exclusions [23,24,25,26]. Three studies clearly described participant-selection criteria and provided complete outcome data [23,25,26]. Three studies reported diabetes duration [24,25,26]. Two studies used ABPI and applied it to both groups, although neither study reported the interval between ultrasound and ABPI assessment or described the ABPI procedure in detail [23,25]. One study reported blinded assessment [23]. All four studies had a low risk of bias [23,24,25,26]. Three studies had a low risk related to group classification [23,25,26], whereas one had moderate risk [24]. Moderate concerns regarding confounding and participant selection were identified in three studies [23,25,26], while these domains had a high risk in one study [24]. The risk associated with deviations or additional care was low in two studies [25,26] and moderate in two [23,24]. Outcome measurement had a low risk in one study [23] and a moderate risk in three studies [24,25,26]. Selective reporting was judged to have a moderate risk in three studies [23,25,26] and a high risk in one study [24]. On the ROBINS-E assessment, no study achieved an overall low risk of bias. Three studies [23,24,25] had an overall high risk of bias, and one study [26] had an overall moderate risk of bias. Bias from exposure measurement, post-exposure interventions, missing data, and selection of the reported result was low in all four studies [23,24,25,26]. The risk of bias due to confounding was moderate in two studies [23,26] and high in two studies [24,25]. The risk of bias in participant selection was low in two studies [25,26] and high in two studies [23,24]. Bias arising from outcome measurement was rated as low in two studies [23,26] and high in two studies [24,25].

## 4. Discussion

This systematic review and meta-analysis examined whether ultrasound-derived morphological and hemodynamic parameters of the lower-limb arteries differ between patients with T2DM and non-diabetic controls. Across four studies comprising 708 participants and 2069 artery-level assessments, neither the overall morphological estimate nor the overall hemodynamic estimate differed significantly between groups. Nevertheless, the parameter-specific findings were not uniformly negative. Wall thickness was greater in patients with T2DM, while flow velocity and flow volume tended to be lower, although the latter differences did not reach statistical significance. In contrast, arterial inner radius, RI, and PI were similar between groups. Collectively, these findings suggest that T2DM may be associated with early structural and flow-related abnormalities, but the available evidence comparing T2DM patients and non-diabetic controls is limited and heterogeneous.

Chronic hyperglycemia, which reduces nitric oxide bioavailability and increases oxidative stress, promotes endothelial dysfunction and a pro-inflammatory, pro-thrombotic vascular environment [27,28,29]. Advanced glycation end-products further impair arterial elasticity by cross-linking extracellular-matrix proteins and promoting vascular smooth-muscle-cell proliferation [30,31]. Shifting smooth muscle cells toward a synthetic phenotype increases extracellular-matrix deposition and intimal hyperplasia and provides a mechanistic basis for arterial wall thickening in T2DM [30,31,32]. Subgroup analysis of wall thickness in the present study demonstrated an increase in arterial wall thickness in patients with T2DM, but not in inner radius or arterial diameter. Conversely, Jiang and Zhao (2017) reported smaller artery diameters in patients with T2DM [24]. This may reflect differences in coexisting cardiovascular diseases. In their cohort, 87.6% of participants had arterial plaques, indicating more advanced disease than in the other studies in which Zou et al. (2017) excluded overt peripheral arterial disease [23], Ali et al. (2022) studied well-controlled, asymptomatic patients with normal ABPI [25], and Zhao et al. (2026) excluded stenosis ≥50% and focused on early popliteal arterial changes [26]. Furthermore, Ali et al. (2022) reported larger arterial diameters in patients with T2DM than in controls on grey-scale imaging, whereas color Doppler measurements of luminal diameter did not differ significantly between the groups [25]. This discrepancy may reflect differences in measurement technique. Color Doppler is sensitive to color gain, pulse-repetition frequency, wall-filter settings, spatial resolution, and incomplete color filling in small distal vessels. Estimates may also vary according to measurement boundaries, cardiac-cycle timing, transducer frequency, arterial segment, and the inclusion of focal plaques. It is important to consider that arterial remodeling encompasses changes in wall thickness, vessel inner radius and diameter that do not necessarily occur in parallel. The increased wall thickness may reflect intimal–medial hypertrophy, extracellular-matrix accumulation, or calcification, whereas vessel inner radius and diameter may depend on the balance between wall thickening, the presence of stenotic plaque and compensatory remodeling [33,34].

The overall hemodynamic analysis showed no significant difference in lower-limb arterial parameters between patients with T2DM and non-diabetic controls. However, the substantial heterogeneity suggests that hemodynamic alterations may depend on the patient characteristics and the arterial segment examined. Zou et al. (2017) reported lower flow velocities in patients with T2DM, potentially reflecting impaired vasodilatory capacity, increased arterial stiffness, elevated downstream resistance, or reduced microvascular recruitment [23]. Conversely, Jiang and Zhao (2017) reported higher PSV and lower flow volumes [24]. This pattern may indicate flow acceleration through a narrowed segment, demonstrated with elevated PSV and coexisting reduced flow volume and impaired perfusion. Diffuse disease without focal stenosis may produce globally reduced velocity and volume flow; thus, PSV should be interpreted alongside vessel morphological characteristics and sampling location. In addition, although the pooled RI and PI estimates were also non-significant, these indices may remain unchanged when systolic and diastolic velocities decline proportionally, and their heterogeneity across studies may reflect differences in arterial compliance, distal resistance, collateral circulation, and spectral waveform acquisition.

Across the included studies, morphological changes in the proximal and knee-level arteries were small and inconsistent, with no significant differences between patients with T2DM and non-diabetic controls [23,24,25,26]. In only one study, the left, but not the right, popliteal artery was significantly smaller [24]. In contrast, abnormalities were more consistently observed in the distal infrapopliteal circulation, with significantly smaller dorsalis pedis artery diameters in T2DM and greater wall thickness in the posterior tibial and dorsalis pedis arteries [24,25]. Similarly, hemodynamics in proximal arteries largely overlapped between groups, whereas more pronounced abnormalities were observed distally [23,24]. These findings suggest that diabetes-related morphological and hemodynamic changes may be more evident below the knee than in proximal segments, consistent with the predominantly distal distribution of diabetic lower-limb arterial disease. It has been reported that patients with diabetes are significantly less likely to have aortoiliac disease, but more likely to have tibial artery disease compared to non-diabetic patients [35,36]. Angiographic data demonstrate this distal predilection, with one cohort of 417 diabetic subjects with critical limb ischemia showing 74% of lesions located in below-the-knee arteries [37]. Comparative angiography further reveals that non-diabetics exhibit more severe femoropopliteal disease, while diabetics show significantly greater infrapopliteal involvement, particularly in the anterior tibial artery [38]. The non-significant pooled morphological estimate may therefore reflect opposing, site-specific remodeling patterns rather than an absence of diabetic vascular morphological change, highlighting the need for independent validation and site-specific reference values.

Four comparative studies were eligible and were single-center and cross-sectional. The included cohorts represented markedly different clinical stages, ranging from well-controlled, asymptomatic T2DM without established PAD to longer-standing disease accompanied by arterial wall thickening, plaque, or stenotic lesions. Differences in age, diabetes duration, glycemic control, renal function, medication use, smoking, and other cardiovascular risk factors may have attenuated or exaggerated the pooled effects. Furthermore, inconsistent reporting of PAD severity precluded subgroup analysis by disease stage, limiting the interpretation and generalizability of the pooled findings. Parameter-specific analyses of wall thickness and inner radius were each derived from a single study contributing one or more artery-level estimates and should therefore be interpreted as structured presentations of the available evidence. Control groups also varied from strictly screened healthy individuals to participants with plaque or stenosis. Consequently, the pooled null findings may reflect clinical-spectrum and participant-selection effects rather than an absence of diabetes-associated vascular injury. In addition, methodological heterogeneity, including differences in arterial segments, ultrasound systems, acquisition settings and measurement methods, was present. The observation that diabetes-related vascular abnormalities may be more pronounced in distal below-knee arteries is based on a limited number of heterogeneous studies evaluating different arterial segments. This apparent distal predominance therefore requires confirmation in larger studies using standardized, segment-specific assessment protocols. Furthermore, although the ultrasound parameters were organized into morphological and hemodynamic domains, the measurements within each domain represent distinct biological and physiological characteristics. The standardized mean difference allows outcomes measured on different scales to be combined statistically but does not imply that they represent the same biological or physiological construct. Therefore, the domain-level estimates have limited standalone clinical interpretability and should be regarded as exploratory. The meta-analysis used the artery-level counts reported by the primary studies as sample sizes because participant-level data and within-participant correlation estimates were unavailable. Although group sample sizes were apportioned across multiple parameters within a forest plot, this did not account for correlation between arteries or arterial segments measured in the same participant. Treating these assessments as independent may have underestimated standard errors and assigned excessive weight to studies contributing multiple assessments per participant. The pooled estimates provide an exploratory summary of the available evidence; however, their confidence intervals and *p*-values warrant cautious interpretation because unaccounted within-participant correlation may affect the precision and weighting of the study estimates. The very high heterogeneity in the morphological and hemodynamic analyses (I^2^ = 93% and 83%, respectively) further limits interpretation, given the small number of included studies. Accordingly, the absence of statistically significant pooled effects should not be interpreted as evidence of an absence of vascular differences between groups. Only one included study reported sensitivity and specificity of the flow velocity parameter. Because only four studies were included, funnel-plot analysis would not provide a reliable assessment of publication bias and was therefore not performed. Future multicentre studies should use standardized ultrasound protocols, clearly defined arterial segments and outcome domains, adjustment for major confounders, and above- and below-knee methods that account for correlated measurements, and establish and validate diagnostic thresholds of ultrasound-derived parameters. This standardization is essential in establishing diagnostic thresholds for ultrasound-derived parameters.

## 5. Conclusions

Although the pooled overall morphological and hemodynamic parameters showed no statistically significant difference between patients with T2DM and non-diabetic controls, arterial abnormalities reported in distal infrapopliteal arteries suggest that diabetes-related vascular changes may be parameter- and segment-specific. These findings support the combined assessment of arterial wall thickness, vessel diameter, flow velocity and volume rather than reliance on a single conventional ultrasound parameter. This approach may be particularly valuable in asymptomatic patients or those with preserved ABPI values, in whom early distal vascular abnormalities may otherwise remain undetected. However, the available evidence is limited by four single-center cross-sectional studies, heterogeneous imaging methods, and correlated artery-level measurements. With further methodological standardization, conventional ultrasound combined with advanced ultrasound imaging technologies such as wall-shear-stress assessment and shear-wave elastography may improve the detection and characterization of diabetes-associated vascular dysfunction. Future prospective multicentre studies with longitudinal follow-up are warranted to establish segment-specific reference values, determine clinically meaningful thresholds, and evaluate the prognostic value of these ultrasound parameters.

## Figures and Tables

**Table 1:** Summary of included studies in chronological order.

Reference (Author/Year)	Aim	Total No. of Participants (T2DM/Control)	Arteries Assessed	Sample Size (T2DM/Control)	Mean Duration of T2DM (Years)	ABPI as Reference	Ultrasound Assessment (Morphology/Hemodynamics)	Findings
Zou et al. (2017) [23]	To explore the clinical value of Ultrasound Doppler in assessing Blood flow to foot and toe	106 (56/50)	RT DPA and PDA	212 (112/100)	-	Yes	Hemodynamics	Patients with T2DM had significantly lower flow velocity, but significantly higher PI and RI than controls.
Jiang and Zhao (2017) [24]	To explore the clinical significance of ultrasound imaging in diagnosing lower extremity vascular disease	131 (81/50)	Bilateral FA, Pop A, and DPA	786 (486/300)	11.58	No	Morphology and hemodynamics	T2DM patients had smaller arterial diameters and altered hemodynamics. Atherosclerotic plaques were more frequent in T2DM, predominantly affecting vessels below the popliteal artery.
Ali et al. (2022) [25]	To assess morphological and hemodynamic parameters of arteries supplying the foot via ultrasound	200 (100/100)	Bilateral PTA and DPA	800 (400/400)	10.35	Yes	Morphology and hemodynamics	Arterial diameter and wall thickness were greater in patients with T2DM than in controls, whereas RI and PI did not differ significantly between the groups.
Zhao et al. (2026) [26]	To establish a marker of lower extremity arterial disease	271 (202/69)	Pop A	271 (202/69)	11.63	No	Morphology and hemodynamics	T2DM group showed a significant reduction in flow velocity, with no significant differences in inner lumen radius compared to control.

Abbreviation: DPA, dorsalis pedis artery; FA, femoral artery; PDA, plantar digital artery; PI, pulsatility index; Pop A, popliteal artery; PTA, posterior tibial artery; RI, resistive index; RT, right; T2DM, type 2 diabetes mellitus.

**Table 2:** Ultrasound morphological parameters of the lower-limb arteries in patients with T2DM and non-diabetic controls.

Reference (Author/Year)	Diabetic Mean ± SD	Non-Diabetic Mean ± SD	*p*-Value
Wall thickness (mm)			
Ali et al. (2022) [25]_RT PTA	0.58 ± 0.15	0.38 ± 0.08	<0.001
Ali et al. (2022) [25]_LT PTA	0.56 ± 0.13	0.38 ± 0.08	
Ali et al. (2022) [25]_RT DPA	0.58 ± 0.15	0.38 ± 0.08	
Ali et al. (2022) [25]_LT DPA	0.56 ± 0.12	0.38 ± 0.08	
Inner radius (mm)			
Zhao et al. (2026) [26]	4.75 ± 0.89	4.56 ± 0.79	0.114
Vessel diameter (mm)			
Jiang and Zhao (2017) [24]_RT FA	7.11 ± 0.89	7.21 ± 0.81	>0.05
Jiang and Zhao (2017) [24]_LT FA	6.87 ± 1.19	7.42 ± 0.69	
Jiang and Zhao (2017) [24]_RT Pop A	5.38 ± 0.59	5.79 ± 0.49	
Jiang and Zhao (2017) [24]_LT Pop A	5.27 ± 0.96	5.86 ± 0.65	<0.05
Jiang and Zhao (2017) [24]_RT DPA	1.21 ± 0.69	1.94 ± 0.39	
Jiang and Zhao (2017) [24]_LT DPA	1.39 ± 0.58	1.86 ± 0.19	
Ali et al. (2022) [25]_RT PTA GS	2.70 ± 0.46	2.49 ± 0.31	<0.001
Ali et al. (2022) [25]_RT PTA CD	1.97 ± 0.33	1.99 ± 0.29	0.588
Ali et al. (2022) [25]_LT PTA GS	2.67 ± 0.45	2.42 ± 0.34	<0.001
Ali et al. (2022) [25]_LT PTA CD	1.93 ± 0.33	1.93 ± 0.25	0.981
Ali et al. (2022) [25]_RT DPA GS	2.81 ± 0.44	2.64 ± 0.36	0.003
Ali et al. (2022) [25]_RT DPA CD	2.03 ± 0.37	2.04 ± 0.29	0.754
Ali et al. (2022) [25]_LT DPA GS	2.90 ± 0.49	2.63 ± 0.31	<0.001
Ali et al. (2022) [25]_LT DPA CD	2.05 ± 0.38	2.03 ± 0.29	0.666

Abbreviation: CD, color Doppler; DPA, dorsalis pedis artery; FA, femoral artery; GS, grayscale; LT, left; Pop A, popliteal artery; PTA, posterior tibial artery; RT, right.

**Table 3:** Ultrasound hemodynamic parameters of the lower-limb arteries in patients with T2DM and non-diabetic controls.

Reference (Author/Year)	Diabetic Mean ± SD	Non-Diabetic Mean ± SD	*p*-Value
Flow velocity (cm/s)			
Zou et al. (2017) [23]_RT DPA PSV	32.02 ± 8.28	38.67 ± 7.59	<0.05
Zou et al. (2017) [23]_RT PDA PSV	24.33 ± 5.22	30.20 ± 4.27	
Zou et al. (2017) [23]_RT DPA MV	10.55 ± 2.08	16.24 ± 2.97	
Zou et al. (2017) [23]_RT PDA MV	8.78 ± 2.12	14.54 ± 2.77	
Zou et al. (2017) [23]_RT DPA EDV	4.42 ± 1.18	6.34 ± 1.79	
Zou et al. (2017) [23]_RT PDA EDV	3.68 ± 0.56	4.45 ± 0.86	
Jiang and Zhao (2017) [24]_RT FA PSV	90 ± 13	87 ± 11	>0.05
Jiang and Zhao (2017) [24]_LT FA PSV	99 ± 19	86 ± 21	
Jiang and Zhao (2017) [24]_RT Pop A PSV	68 ± 50	56 ± 15	
Jiang and Zhao (2017) [24]_LT Pop A PSV	65 ± 21	52 ± 11	<0.05
Jiang and Zhao (2017) [24]_RT DPA PSV	61 ± 27	37 ± 12	
Jiang and Zhao (2017) [24]_LT DPA PSV	45 ± 16	37 ± 14	
Zhao et al. (2026) [26]_Pop A PSV	51.31 ± 14.81	65.35 ± 13.94	<0.05
Flow volume (mm^3^/s)			
Jiang and Zhao (2017) [24]_RT FA	36.49 ± 15.97	34.29 ± 6.97	>0.05
Jiang and Zhao (2017) [24]_LT FA	37.21 ± 12.98	40.31 ± 6.94	
Jiang and Zhao (2017) [24]_RT Pop A	12.65 ± 3.98	13.70 ± 2.36	
Jiang and Zhao (2017) [24]_LT Pop A	13.95 ± 6.25	13.58 ± 2.98	
Jiang and Zhao (2017) [24]_RT DPA	0.71 ± 0.49	1.42 ± 0.64	<0.05
Jiang and Zhao (2017) [24]_LT DPA	0.75 ± 0.59	1.25 ± 0.49	
RI			
Zou et al. (2017) [23]_RT DPA	0.93 ± 0.15	0.84 ± 0.14	<0.05
Zou et al. (2017) [23]_RT PDA	0.94 ± 0.13	0.88 ± 0.16	
Ali et al. (2022) [25]_RT PTA	0.93 ± 0.13	0.93 ± 0.08	0.577
Ali et al. (2022) [25]_LT PTA	0.94 ± 0.14	0.93 ± 0.09	0.574
Ali et al. (2022) [25]_RT DPA	0.94 ± 0.13	0.93 ± 0.08	0.418
Ali et al. (2022) [25]_LT DPA	0.93 ± 0.13	0.93 ± 0.08	0.574
PI			
Zou et al. (2017) [23]_RT DPA	2.69 ± 0.41	2.44 ± 0.36	*p* < 0.05
Zou et al. (2017) [23]_RT PDA	2.51 ± 0.39	2.26 ± 0.33	
Ali et al. (2022) [25]_RT PTA	8.49 ± 2.63	8.61 ± 2.47	0.730
Ali et al. (2022) [25]_LT PTA	8.59 ± 2.63	8.57 ± 2.47	0.951
Ali et al. (2022) [25]_RT DPA	8.50 ± 2.92	8.40 ± 2.39	0.810
Ali et al. (2022) [25]_LT DPA	8.65 ± 2.72	8.62 ± 2.48	0.929

Abbreviation: DPA, dorsalis pedis artery; EDV, end-diastolic velocity; FA, femoral artery; LT, left; MV, mean velocity; PDA, plantar digital artery; PI, pulsatility index; Pop A, popliteal artery; PSV, peak systolic velocity; PTA, posterior tibial artery; RI, resistive index; RT, right.

## Data Availability

The data presented in this study are included in the article. Further inquiries can be directed to the corresponding author.

## Supplementary Materials

The following supporting information can be downloaded at https://www.mdpi.com/article/10.3390/jcm15197580/s1, Table S1: PRISMA 2020 Checklist; Table S2: Boolean search strategy for each database.
