# Supplementary material for: Ultrasound Evaluation of Lower-Limb Arterial Morphology and Hemodynamics in Type 2 Diabetes Mellitus Versus Non-Diabetic Controls: A Systematic Review and Meta-Analysis
Source: J Clin Med. 2026 Sep 29;15(19):7580. doi: 10.3390/jcm15197580 (PMC13666381; doi:10.3390/jcm15197580)
Supplement: Supplementary file 1 [file jcm-15-07580-s001.zip › jcm-4553246-supplementary/Table S2.pdf]

Table S2: Boolean search strategy for each database

Embase

```
((((((((diabetes AND mellitus OR diabetic) AND foot OR diabetic OR type) AND 2 AND diabetes OR type) AND 1 AND diabetes OR healthy) AND volunteers OR 'non diabetic') AND controls OR healthy) AND controls OR control) AND group OR control) AND groups OR healthy) AND participants AND ((((((doppler AND ultrasound OR duplex) AND ultrasound OR doppler) AND ultrasonography OR colour) AND doppler OR color) AND doppler OR spectral) AND doppler OR vascular) AND ultrasound OR arterial) AND ultrasound AND ((((((((((peak AND systolic AND velocity OR 'end diastolic') AND velocity OR resistive) AND index OR pulsatility) AND index OR acceleration) AND time OR 'intima media') AND thickness OR flow) AND volume OR arterial) AND diameter OR blood) AND flow AND velocity OR blood) AND flow AND volume OR ankle) AND brachial AND index OR 'ankle brachial') AND index AND (((((((lower AND extremity OR leg OR femoral) AND artery OR popliteal) AND artery OR tibial) AND artery OR lower) AND limb OR dorsalis) AND pedis OR posterior) AND tibial OR anterior) AND tibial
```

Cochrane

```
(diabetes mellitus OR diabetic foot OR diabetic OR type 2 diabetes OR type 1 diabetes OR healthy volunteers OR non-diabetic controls OR healthy controls OR control group OR control groups OR healthy participants) AND (doppler ultrasound OR duplex ultrasound OR doppler ultrasonography OR colour doppler OR color doppler OR spectral doppler OR vascular ultrasound OR arterial ultrasound) AND (peak systolic velocity OR end-diastolic velocity OR resistive index OR pulsatility index OR acceleration time OR intima-media thickness OR flow volume OR arterial diameter OR blood flow velocity OR blood flow volume OR ankle brachial index OR ankle-brachial index) AND (lower extremity OR leg OR femoral artery OR popliteal artery OR tibial artery OR lower limb OR dorsalis pedis OR posterior tibial OR anterior tibial)
```

Web of science

```
(diabetes mellitus OR diabetic foot OR diabetic OR type 2 diabetes OR type 1 diabetes OR healthy volunteers OR non-diabetic controls OR healthy controls OR control group OR control groups OR healthy participants) AND (doppler ultrasound OR duplex ultrasound OR doppler ultrasonography OR colour doppler OR color doppler OR spectral doppler OR vascular ultrasound OR arterial ultrasound) AND (peak systolic velocity OR end-diastolic velocity OR resistive index OR pulsatility index OR acceleration time OR intima-media thickness OR flow volume OR arterial diameter OR blood flow velocity OR blood flow volume OR ankle brachial index OR ankle-brachial index) AND (lower extremity OR leg OR femoral artery OR popliteal artery OR tibial artery OR lower limb OR dorsalis pedis OR posterior tibial OR anterior tibial)
```

PubMed

```
( "Diabetes Mellitus"[MeSH Terms] OR "Diabetic Foot"[MeSH Terms] OR diabet* OR "Type 2 Diabetes Mellitus"[MeSH Terms] OR "Type 1 Diabetes Mellitus"[MeSH Terms] OR "healthy volunteers"[MeSH Terms] OR non-diabetic control* OR healthy control* OR control group* OR healthy participant* ) AND ( "Ultrasonography, Doppler"[MeSH Terms] OR "Ultrasonography, Doppler, Duplex"[MeSH Terms] OR "Ultrasonography, Doppler, Color"[MeSH Terms] OR "Ultrasonography, Doppler, Pulsed"[MeSH Terms] OR duplex ultrasound OR colour Doppler OR color Doppler OR spectral Doppler OR vascular ultrasound OR arterial ultrasound ) AND ( "peak systolic velocity" OR "end-diastolic velocity" OR "resistive index" OR "pulsatility index" OR "acceleration time" OR "intima-media thickness" OR "flow volume" OR "arterial diameter" OR "blood flow velocity" OR "blood flow volume" OR "ankle brachial index" OR "ankle-brachial index" ) AND ( "Lower Extremity"[MeSH Terms] OR "Leg"[MeSH Terms] OR "Femoral Artery"[MeSH Terms] OR "Popliteal Artery"[MeSH Terms] OR "Tibial Arteries"[MeSH Terms] OR lower limb OR lower extremity OR femoral artery OR popliteal artery OR tibial artery OR dorsalis pedis OR posterior tibial OR anterior tibial )
```
